# Editorial: Thermophilic and halophilic extremophiles in Eurasian environments, volume II

**DOI:** 10.3389/fmicb.2023.1160288

**Published:** 2023-02-28

**Authors:** Hongchen Jiang, Wen-Jun Li, Nils-Kåre Birkeland, Dilfuza Egamberdieva

**Affiliations:** ^1^State Key Laboratory of Biogeology and Environmental Geology, China University of Geosciences, Wuhan, China; ^2^State Key Laboratory of Desert and Oasis Ecology, Xinjiang Institute of Ecology and Geography, Chinese Academy of Sciences, Urumqi, China; ^3^State Key Laboratory of Biocontrol and Guangdong Provincial Key Laboratory of Plant Resources, School of Life Sciences, Sun Yat-sen University, Guangzhou, China; ^4^Department of Biological Sciences, University of Bergen, Bergen, Norway; ^5^Institute of Fundamental and Applied Research, National Research University Tashkent Institute of Irrigation and Agricultural Mechanization Engineers, Tashkent, Uzbekistan; ^6^Faculty of Biology, National University of Uzbekistan, Tashkent, Uzbekistan

**Keywords:** saline/hypersaline environments, thermal environments, microbial diversity, adaptation, pathways

Saline/hypersaline and geothermal ecosystems are typical extreme environments. Microbial diversity, ecological functions and adaptations in (hyper)saline and thermal ecosystems are receiving extensive attention, mainly because: (1) they have environmental conditions similar to that of some extreme environments on early Earth or other planets, so they are suitable environments for simulation research on the origin and evolution of life and the exploration of extraterrestrial life; (2) they have relatively low complexity of microbial communities, so they are often employed as model ecosystems to investigate microbially mediated element cycling and biogeochemical processes and their responses to environmental conditions (e.g., salinity, temperature); and (3) they are rich in microbial dark matter (a large number of microorganisms existing in nature that cannot be cultivated in the laboratory and are hardly known), so they are employed to obtain new microbial resources and discover new metabolic pathways.

The saline/hypersaline and geothermal ecosystems in Eurasia have diverse and unique geological and physiochemical characteristics. Multi-omics techniques have enabled a series of new discoveries in microbial diversity, ecological functions and biogeochemistry of these environments. In 2019, we successfully organized and published one Research Topic consisting of 11 original research articles (https://www.frontiersin.org/research-topics/5339/thermophilic-and-halophilic-extremophiles-in-eurasian-environments) with the same title as the present one, which received a good reading feedback (7,000 downloads and 37,000 views) in the year after its publication. Therefore, Chief Editor Andreas Teske invited us to relaunch the Research Topic. We gladly accepted his invitation to organize this Research Topic.

In the current Research Topic, we accepted and published eight research articles, including five and three articles on saline/hypersaline and geothermal ecosystems, respectively. We are grateful to all authors who contributed to this Research Topic. We are also grateful to all reviewers, handling editors and editorial staff who contributed during the editing and article production processes.

Among the articles related to studies on saline/hypersaline ecosystems, Singh et al. disclosed that elevated inorganic carbon and salinity enhances photosynthesis and ATP synthesis in picoalga *Picocystis salinarum* as revealed by label free quantitative proteomics; Liu Q. et al. found that the diversity of carbohydrate-active enzymes (CAZy) and sulfur cycling genes decreased with increasing salinity, whereas nitrogen cycling gene diversity showed an opposite trend. Relative abundances of many CAZy, nitrogen and sulfur cycling gene categories decreased with increasing salinity, whereas some CAZy, nitrogen and sulfur gene categories showed an increasing trend. The compositions of CAZy, nitrogen, and sulfur cycling genes in the studied lake sediments were significantly (*P* < 0.05) affected by environmental factors such as salinity, total organic carbon, total nitrogen, and total phosphorus, with salinity having the greatest influence. Liu M. et al. identified the biosynthetic pathway of glycine betaine that is responsible for salinity tolerance in halophilic *Thioalkalivibrio versutus* D301. Yi et al. characterized and analyzed the genome of a novel halovirus infecting *Chromohalobacter beijerinckii*. Lin et al. found that rare taxa drive the response of soil fungal guilds to soil salinization in the Taklamakan Desert.

Among the articles related to studies on geothermal ecosystems, Song et al. characterized the *nifH* gene expression and diversity in geothermal springs of Tengchong, China. Yuan et al. described different regulatory strategies of arsenite oxidation by two *Thermus tengchongensis* strains isolated from hot springs. Khomyakova et al. reported the first cultivated representatives, constituting a novel order *Anaerosomatales*.

We are delighted to publish this Research Topic in Frontiers in Microbiology. We hope that this Research Topic will be interesting and useful to the readers of the journal, and broaden the knowledge of thermophilic and halophilic extremophiles in Eurasian Environments. The findings presented in this Research Topic are exciting, but still limited. In the future, the application of innovative research technologies and intensive and in-depth international collaboration will undoubtedly unveil more exciting aspects of thermophilic and halophilic extremophiles in Eurasian environments.

## Author contributions

HJ organized this topic and wrote the editorial article. W-JL, N-KB, and DE are co-editors of the topic and discussed the writing. All authors contributed to the article and approved the submitted version.

